# Genome Analysis of a Newly Discovered Yeast Species, *Hanseniaspora menglaensis*

**DOI:** 10.3390/jof10030180

**Published:** 2024-02-28

**Authors:** Adam P. Ryan, Marizeth Groenewald, Maudy Th. Smith, Cian Holohan, Teun Boekhout, Kenneth H. Wolfe, Geraldine Butler

**Affiliations:** 1 School of Biomolecular and Biomedical Science, Conway Institute, University College Dublin, Belfield, D04 V1W8 Dublin 4, Ireland; cianholohan@gmail.com (C.H.); gbutler@ucd.ie (G.B.); 2 Westerdijk Fungal Biodiversity Institute, 3584 CT Utrecht, The Netherlands; m.groenewald@wi.knaw.nl (M.G.); m.smith@wi.knaw.nl (M.T.S.); 3 College of Sciences, King Saud University, P.O. Box 2455, Riyadh 11451, Saudi Arabia; teun.boekhout@gmail.com; 4 School of Medicine, Conway Institute, University College Dublin, Belfield, D04 V1W8 Dublin 4, Ireland; kenneth.wolfe@ucd.ie

**Keywords:** heterothallic, MAT locus, sporulation, yeast mitochondrion, phylogenomics, nanopore sequencing, Illumina sequencing, genome assembly, chromosomes, fast-evolving lineage

## Abstract

Annual surveys of Irish soil samples identified three isolates, CBS 16921 (UCD88), CBS 18246 (UCD443), and CBS 18247 (UCD483), of an apiculate yeast species within the *Hanseniaspora* genus. The internal transcribed spacer (ITS) and D1/D2 region of the large subunit (LSU) rRNA sequences showed that these are isolates of the recently described species *Hanseniaspora menglaensis*, first isolated from Southwest China. No genome sequence for *H. menglaensis* is currently available. The genome sequences of the three Irish isolates were determined using short-read (Illumina) sequencing, and the sequence of one isolate (CBS 16921) was assembled to chromosome level using long-read sequencing (Oxford Nanopore Technologies). Phylogenomic analysis shows that *H. menglaensis* belongs to the fast-evolving lineage (FEL) of *Hanseniaspora*. Only one *MAT* idiomorph (encoding *MAT*α1) was identified in all three sequenced *H. menglaensis* isolates, consistent with one mating type of a heterothallic species. Genome comparisons showed that there has been a rearrangement near *MAT*α of FEL species compared to isolates from the slowly evolving lineage (SEL).

## 1. Introduction

*Hanseniaspora* species are apiculate yeasts found abundantly on a variety of ripening fruits, flowers and barks [1]. They are particularly prevalent and diverse within grape musts [2,3]. *Hanseniaspora* species have long since been associated with wine fermentation, commonly as pests, and more recently as potential bio-flavouring agents [3,4]. Most species are not prolific fermenters with low ethanol tolerance between 3 and 5% and are quickly outcompeted by *Saccharomyces* species in early fermentation [5,6]. Those that do exceed this threshold, such as *Hanseniaspora osmophila*, often produce “off flavour” compounds such as acetic acid, acetaldehyde, and ethyl acetates, which are considered detrimental to the flavour profile of the wine [7,8]. *Hanseniaspora* species may function as bio-flavouring agents and potential co-fermenters with *Saccharomyces cerevisiae* because they can metabolise cellobiose [9,10].

*Hanseniaspora* species fall into two subclades commonly known as the fast (FEL) and slow (SEL) evolving lineages within the *Saccharomycodaceae* [11]. The clades are distinguished by the loss of genes involved in DNA repair, cell cycle repair, and mitotic checkpoints [11], with more extensive loss observed within the FEL. The loss of repair genes enabled the rapid accumulation of mutations, resulting in significant protein divergence. The FEL is separated from other lineages by a distinctly long branch length, similar to many hyper-mutator fungal lineages [11]. Members of the SEL are more proficient fermenters, yielding higher concentrations of ethanol [12].

Currently, 24 species of *Hanseniaspora* are known, consisting of 18 species within the FEL and 6 within the SEL [13]. Three yeast strains, CBS 16921 (UCD88), CBS 18246 (UCD443), and CBS 18247 (UCD483), were collected from soil samples as part of undergraduate projects at University College Dublin, Ireland, from 2017 to 2020 [14,15,16]. Strains were isolated from Ballawley Park, Co. Dublin (53.2799, −6.23317) (CBS 16921) and wooded roadsides in Kilcommon, Co. Tipperary (52.692835, −8.146708) (CBS 18247) and Aughnagarnon, Co. Longford (53.788433, −7.423251) (CBS 18246). Internal transcribed spacer (ITS) sequence analysis suggests they represent new isolates of the recently described species *Hanseniaspora menglaensis* [13] (Table A1). Because there is currently no genome sequence of *H. menglaensis* available, we sequenced the genomes of the three Irish isolates in order to contribute to our understanding of the phylogeny and evolution of the *Hanseniaspora* genus.

## 2. Materials and Methods

**Yeast isolation and identification**: Yeasts were isolated from soil samples as described in Sylvester et al. [17] and Bergin et al. [16]. In brief, ~2.5 g of soil was incubated at room temperature for 5 days in 9 mL yeast–peptone–dextrose (YPD) (1% yeast extract, 2% peptone, 2% glucose) broth containing chloramphenicol (30 μg/mL) and ampicillin (100 μg/mL). A total of 10 µL of homogenised cultures was inoculated into fresh media for a further 2-day incubation. Next, 100 µL of both 1:100 and 1:10,000 diluted cultures was plated onto YPD agar (1% yeast extract, 2% peptone, 2% agar, 2% glucose) and incubated for 5 days. Single colonies were obtained and potential yeast isolates were chosen for further investigation. The ITS regions of selected isolates were amplified by colony PCR using universal primers ITS1 (5′-TCCGTAGGTGAACCTGCGG-3′) and ITS4 (5′-TCCTCCGCTTATTGATATGC-3′) [18] using 35 cycles of 95° for 15 s, 48 °C for 15 s, and 72 °C for 30 s. PCR products were sequenced by the Eurofins Genomics Mix2Seq platform using ITS1 as a primer. Three isolates with ITS sequences identical to the ITS sequence of *Hanseniaspora menglaensis* (CICC 33364/NYNU 181083) were deposited to the Westerdijk Fungal Biodiversity Institute, Utrecht, The Netherlands, as CBS 16921, CBS 18246 and CBS 18247 (Table A1) [13]. Additional deposits have been made to the Portuguese Yeast Culture Collection, Portugal, as PYCC 9756 (CBS 16921), PYCC 9757 (CBS 18246) and PYCC 9758 (CBS 18247) and at University College Dublin as UCD88 (CBS 16921), UCD443 (CBS 18246) and UCD483 (CBS 18247).

**Genome sequencing**: We sequenced the genome of one isolate (CBS 16921) to chromosome level using a combination of long-read (Oxford Nanopore) and short-read (Illumina) technologies, and we used short-read sequencing to survey the genomes of the other two Irish isolates. For Illumina sequencing, total genomic DNA was prepared from all three Irish *H. menglaensis* isolates using phenol–chloroform–isoamyl alcohol (Sigma-Aldrich P3803, Gillingham, Dorset, UK. All three genomes were sequenced by BGI Tech Solutions Co. (Hong Kong, China) from 1 µg of genomic DNA using an Illumina HiSeq 4000 instrument for CBS 16921 and an Illumina HiSeq X for CBS 18246 and CBS 18247. A total of 6.4 million, 6.7 million, and 4 million paired-end reads (2 × 150 bp) were obtained for CBS 16921, CBS 18246 and CBS 18247, respectively. Reads were trimmed using Skewer v. 0.2.2 [19] to minimum mean qualities of 30 and minimum lengths of 35. To increase the proportion of high-molecular-weight DNA for long-read sequencing which facilitates chromosomal-level assembly, genomic DNA was also extracted from CBS 16921 using a Qiagen Genomic Tip 100/G kit according to the manufacturer’s instructions. Genomic DNA (400 ng) was sequenced with MinION technology using the rapid barcoding kit (SQK-RBK004) from Oxford Nanopore Technologies (ONT, Oxford, UK), following the manufacturer’s instructions. Sequencing was performed on a MinION MK1B device (MinKNOW v. 19.05.0) (ONT) using an r9.4.1 chemistry flowcell (FLO-MIN109). Basecalling was performed using guppy v. 16.04.5 (ONT) and reads were demultiplexed using qcat v. 1.1.0 (ONT). This generated 213,571 raw reads, which were reduced to 182,436 reads by filtering with NanoFilt v. 2.8.0 (ONT) to remove reads with quality scores < 7 or lengths of <1 kb. The filtered reads were assembled into 13 contigs using Canu v. 2.2 [20]. The raw assembly was polished with the trimmed Illumina reads using five rounds of correction with NextPolish v. 1.4.1 [21]. Two contigs containing partial arrays of the rDNA locus at one end each were manually joined. Four short, overlapping contigs derived from the mitochondrial genome were removed. The mitochondrial genome was annotated using MITOS2 [22] and trimmed to one copy using bedtools [23]. The *VAR1* gene was identified by BLAST analysis of an unannotated open reading frame. Contig-level assemblies for samples CBS 18246 and CBS 18247 were generated using SPAdes v. 3.14.0 [24]. Only contigs larger than 500 bases with an average coverage greater than 10 were retained. 

**Genome annotation**: Protein-coding sequences in all *Hanseniaspora* genomes were identified using BRAKER3 v. 3.0.2 [25]. Genomes were first soft-masked using both RepeatModeler v.2.0.4 [26] and RepeatMasker v. 4.1.2-p1 [27]. The training set for protein annotation was derived from the OrthoDB Fungi clade partition [28]. Annotation was performed with a lambda parameter of 1 for intron downsampling [25]. For CBS 16921, InterProScan v. 5.61-93.0 [29] was used to further annotate putative protein function using PANTHER, TIGRFAM, PFAM and SUPERFAMILY databases. tRNA-scan v. 2.0.5 [30] was used to annotate tRNAs, and barrnap v. 0.9 [31] was used to annotate rRNAs. In total, 4260 genomic protein-coding genes, 162 tRNAs, and a three-copy array of the rDNA locus were annotated in the CBS 16921 reference assembly.

Trimmed reads for CBS 16921, CBS 18246 and CBS 18247 were mapped to the CBS 16921 reference genome using BWA v. 0.7.17-r1188 [32]. Alignments were sorted and indexed using SAMtools v. 1.10 [33]. Duplicate reads were marked using Picard tools [34]. Variants were called and filtered using BCFtools v. 1.10.2 [35] and VCFtools v. 0.1.16 [36], respectively. Sites missing in any sample, sites with quality < 30, and sites with depth < 15 or >200 were removed. Only single-nucleotide polymorphisms (SNPs) were analysed. This identified 122 variant sites, all of which were manually verified in IGV [37] using BAM alignment to ensure that the sequencing depth matched the surrounding regions. SNP, protein-coding, tRNA, and rRNA annotations were visualised in R using the Circlize v. 0.4.15 [38] package.

**Phylogenomic analysis**: Phylogenomic analysis was performed using single-copy orthologs from the *H. menglaensis* CBS 16921 chromosome-level assembly, short-read assemblies for CBS 18246 and CBS 18247, and 20 scaffold-level assemblies for other *Hanseniaspora* species. Four outgroup species were included (*Saccharomyces cerevisiae* S288C, *Kluyveromyces marxianus* DMKU3-1042, *Wickerhamomyces anomalus* NRRL Y-366-8 and *Cyberlindnera jadinii* NRRL Y-1542). All included *Hanseniaspora* genomes were annotated as described above. Average nucleotide identity (ANI) was determined pairwise between CBS 16921 and all other assemblies using OrthoAni [39]. Single-copy orthologs were identified as described in Steenwyk et al. [11] with some modifications. In brief, putative orthologs were clustered using OrthoMCL [40] with pairing evidence from BLASTP v.2.10.0 [41] searches. BLASTP hits were filtered for E-values ≤ 1 × 10^−10^, percent identities ≥ 30%, and percent match length ≥ 70%. An inflation parameter of 4 was used to cluster putative orthologs with MCL v.14-137 [40]. Unlike Steenwyk et al. [11], only single-copy orthologs were used. In total, 548 single-copy orthologs were identified. Orthologs were aligned using MAFFT v.7.520 [42] with the following parameters: “--op 1.0 --maxiterate 1000 --genafpair”. Alignments were trimmed using trimAl v.1.4.rev15 (http://trimal.cgenomics.org/ (accessed on 14 August 2023)) [43] using the “automated 1” parameter. Trees were calculated for each ortholog alignment using RAxML v.8.2.12 [11,44] with the model of substitution set to PROTGAMMAAUTO and the seed set to “12345”. Ortholog trees where all four outgroup species were not found to be earliest diverging were discarded. Alignments of the remaining 522 orthologs were concatenated, and trees were inferred using RAxML with 100 bootstraps and the PROTGAMMALG model of substitution and a seed of “12345” [44]. The corresponding tree file was visualised in iTOL [45].

**Mating-type locus annotation***:* The mating-type locus and neighboring genes in *H. menglaensis* isolates CBS 16921, CBS 18246 and CBS 18247 were identified using BLASTN and TBLASTN [41] against a dataset of *Saccharomyces cerevisiae* reference proteins. Similar sequences were identified in other *Hanseniaspora* species using BLAST [11,41]. Pairwise identity was calculated using the *H. menglaensis* sequences of *SLA2*, *SUI1*, *MAT*α1, *VPS75*, *YNL247W*, *GNEAS1* and *CWC25* and *MAT***a**2 from *H. valbyensis*.

**Physiological characterization**: Morphology, nutritional growth and additional phenotypic profiles were characterised using standard protocols as described in Kurtzman et al. [46]. Most growth tests were performed at 25 °C, except for fermentation, which was assayed at 20 °C. Growth at 30 °C was assessed on (GYPA-2% glucose, 1% peptone, 0.5% yeast extract, 1.5% agar, pH 6.8). Ascus and ascospore formation were investigated by growing CBS 16921, CBS 18246 and CBS 18247 separately and as mixed cultures on 2% Difco malt extract agar (MEA) (pH 5.5) at 25 °C. Cells were examined daily for up to 7 days. 

## 3. Results

### 3.1. Genome Sequence of H. menglaensis 

An isolate of the new species *Hanseniaspora menglaensis* was recently identified from rotting wood in Southwest China [13]. We identified three isolates from soil in Ireland: CBS 16921, CBS 18246 and CBS 18247. The ITS regions of CBS 16921, CBS 18246, CBS 18247 are 99% identical to *H. menglaensis* CICC 33364/NYNU 181083, with lower similarity (96.7%) to the next closest sequence (*Hanseniaspora lindneri*) (Table A1). Extraction of the D1/D2 regions shows that CBS 16921, CBS 18246, CBS 18247 and *H. menglaensis* CICC 33364/NYNU 181083 share >99% identity (Table A1). Current guidelines (ITS sequence divergence of <2% and D1/D2 divergence < 1%) indicate that CBS 16921, CBS 18246, CBS 18247 and CICC 33364/NYNU 181083 are isolates of the same species, *H. menglaensis*, separate to *H. lindneri* [47,48,49].

**Figure 1:**
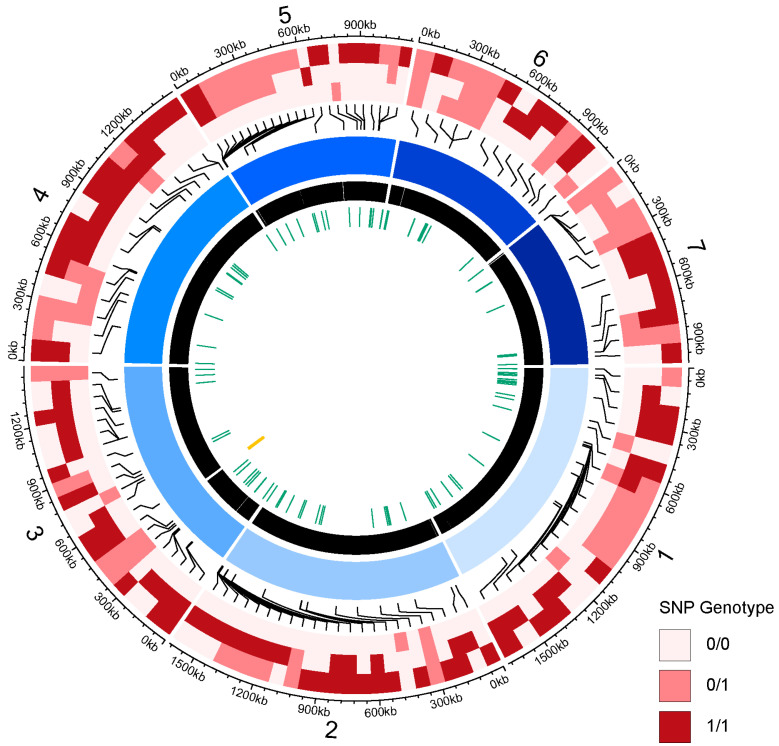
Chromosome circle diagram of *H. menglaensis* CBS 16921 genome assembly. The central circle (blue) shows each chromosome, labelled by number 1 through 7 on the outermost ring. Chromosome sizes are shown in 300 kb intervals. The pink-to-red heatmap rings show the genotypes of well-supported SNPs in comparison to the reference genome, in the order CBS 16921, CBS 18246 and CBS 18247 (from inner to outer). “0/0” represents sites called as homozygous for the reference allele, “1/1” represents sites called as homozygous for an alternative allele, and “0/1” represents sites called as heterozygous. The black ring shows protein-coding sequences, the green ring shows tRNA genes, and the gold ring shows the rRNA array on chromosome 3.

We sequenced the genome of one isolate (*H. menglaensis* CBS 16921) using a combination of long read (Oxford Nanopore, Oxford, UK) and short read (Illumina, Cambridge, UK) technologies, and we used short-read sequencing to survey the genomes of the other two isolates. The final assembly of *H. menglaensis* CBS 16921 consists of 8 contigs (7 chromosome-level contigs, named from 1 to 7 in order of size, and 1 mitochondrial contig) (Figure 1). This assembly is 9,558,052 bp with an N50 of 1,490,982 bp and G+C content of 30.34%. This assembly is likely chromosome-level, but no telomere repeats were identified. The mitochondrial genome consists of a circular contig of 19.63 kb. G+C content is lower than that of the nuclear genome (23.61%). All core mitochondrial components (*rrnL*, *rrnS*, *cob*, *cox1*, *cox2*, *cox3*, *atp6*, *atp8* and *atp9*) are present, as well as 27 tRNA genes. The NADH ubiquinone oxidoreductase genes (*nad1*, *nad2*, *nad3*, *nad4*, *nad4L*, *nad5*, *nad6*) are not present, similar to species in the *Saccharomycetaceae* [50]. The ribosomal protein gene *VAR1* is missing from *Hanseniaspora uvarum* [50,51]. However, it is present in the *H. menglaensis* mitochondrial genome. Approximately 43% of the mitochondrial genome consists of intergenic regions.

Mapping the individual reads from *H. menglaensis* CBS 16921, CBS 18246 and CBS 18247 to the haploid genome assembly identified 122 variant sites (Figure 1). In each isolate, a small number of sites were called as heterozygous with high confidence: there are 24 such sites in CBS 16921, 29 in CBS 18246, and 40 in CBS 18247. In addition, 53 sites in CBS 18246 and 55 sites in CBS 18247 were called as homozygous for an allele different from the reference (the CBS 16921 haploid assembly). The genomes of the three Irish isolates are therefore very similar (~99.987%), but they are not identical (Figure 1). No high-confidence SNPs were identified between the mitochondrial genomes.

**Figure 2:**
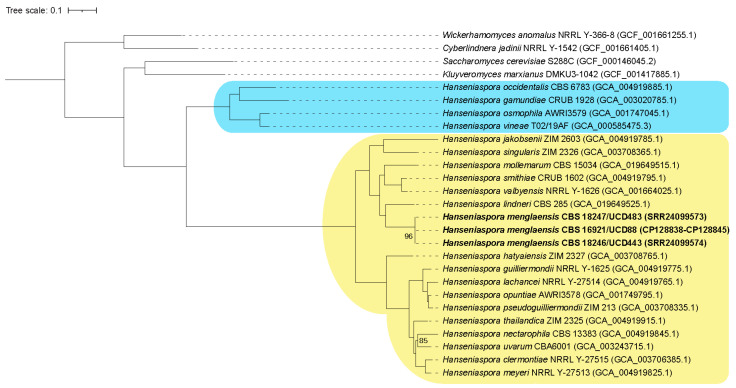
Phylogenomic tree generated from 522 single-copy orthologs from 23 *Hanseniaspora* isolates and 4 outgroup species, *S. cerevisiae*, *K. marxianus*, *W. anomalus* and *C. jadinii*. Bootstraps lower than 100% are shown. The accession of the reference assembly or protein set used is shown in parentheses. The slow (SEL) and fast (FEL) evolving lineages are shown with blue and yellow boxes, respectively. The new species *H. menglaensis* is marked in bold text.

Phylogenomic analysis (Figure 2) shows that *H. menglaensis* CBS 16921, CBS 18246 and CBS 18247 belong to the fast-evolving lineage (FEL) of *Hanseniaspora* and form a subclade with *Hanseniaspora lindneri*, *Hanseniaspora valbyensis*, *Hanseniaspora smithiae*, *Hanseniaspora mollemarum*, *Hanseniaspora singularis* and *Hanseniaspora jakobsenii.* They are most closely related to *H. lindneri* CBS 285 but are separated with bootstrap support of 100%. This tree provides strong support for placing *H. menglaensis* within the FEL as a close relative of *H. lindneri*, similar to a previous analysis which used only the ITS region, D1/D2 domain of the LSU and *ACT1* [13] (bootstrap support of 78%). In addition, comparing the ANI over the entire genome sequences showed that *H. menglaensis* CBS 16921 and *H. lindneri* have an ANI of 75.5% (determined using OrthoAni [39]) (Table A2), supporting the designation of *H. menglaensis* as a separate species.

### 3.2. Characterization of the Mating-Type Locus

Yeast mating occurs as a fusion of haploid cells of opposing mating types [52]. Mating types are determined by idiomorphs of the mating-type (*MAT*) locus, and species may be either heterothallic or homothallic [52,53,54]. Heterothallic species encode one of two *MAT* idiomorphs, *MAT***a** or *MAT*α. Mating occurs between haploid cells of opposite mating type, generating a *MAT***a**/*MAT*α diploid [52,54]. Homothallic species encode mating identity genes from both *MAT***a** and *MAT*α idiomorphs [54,55]. Such isolates can mate with any other related cell [52,53,54,55]. 

**Figure 3:**
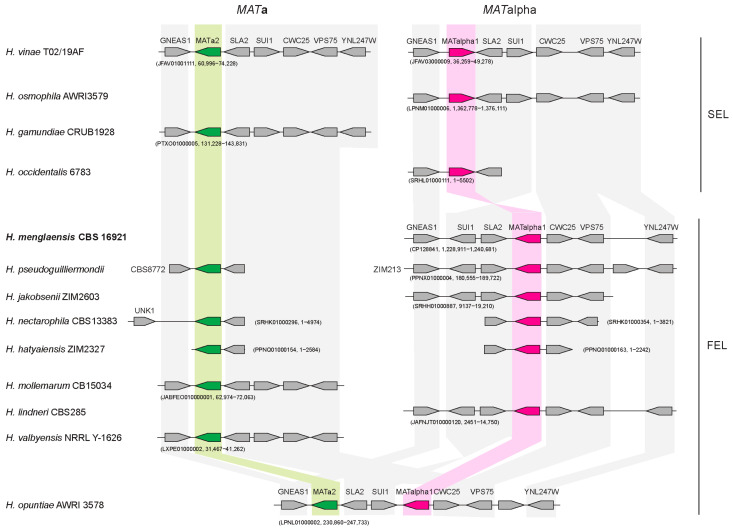
Identification of mating-type (*MAT*) loci. Mating loci were identified using BLAST analysis. Orthologous genes are indicated with shading in the background. *MAT***a**2 genes are colored in green and orthologs are indicated with green shading, whereas *MAT*α1 genes are colored in pink and orthologs are indicated with pink shading. The accession numbers and locations of the *MAT* loci are indicated. *MAT***a** and *MAT*α loci were identified in different assemblies of *H. vinae* TO2/19AF [56]. Some *MAT* loci are assembled into short contigs (e.g., *H. nectarophila* and *H. hatyaiensis*). The *MAT***a** loci of *H. pseudoguilliermondii* CBS8772 and *H. opuntiae* AWRI 357 are described in Saubin et al. [56]. There is a rearrangement around *SUI1/MAT*α1 in FEL isolates, as previously described by Saubin et al. [56]. The three *H. menglaensis* isolates have identical *MAT*α loci. Pairwise similarity with the reference *H. menglaensis* sequence varies for each gene: *CWC25* (34–61.5%), *GNEAS1* (35.4–67.4%), *MAT*α1 (34.6–52.9%), *SLA2* (10.8–76%), *SUI1* (10.1–89.7%), *VPS75* (41.1–77.3%) and *YNL247W* (63.5–80.7%). *MAT***a**2 was compared to *H. valbyensis* and ranges from 37.6 to 65.7% identity.

Heterothallic isolates of *Hanseniaspora* species have previously been identified from both haploid and diploid genome assemblies [55]. The *MAT***a** and *MAT*α idiomorphs encode only one gene each: *MAT***a**2 and *MAT*α1, respectively [53,54,55,56]. Both idiomorphs are found adjacent to *SLA2*, *SUI1*, *CWC25* and *GNEAS1* [56]. However, previous analysis has shown that that there is an inversion between *SUI1* and *MAT*α1 in *H. pseudoguillermondii*, *H. opuntiae*, *H. uvarum* and *H. guilliermondii* (Figure 3) [56].

We used BLASTN and TBLASTN [41] to extend the analysis of the *MAT* locus across 13 *Hanseniaspora* species, including species from both the FEL and SEL (Figure 3). Only one *MAT* idiomorph (encoding *MAT*α1) was identified in all three Irish *H. menglaensis* isolates (Figure 3). The structure of the region resembles the *MAT*α locus in other FEL isolates, with *MAT*α1 lying between *SLA2* and *CWC25* (Figure 3). We find that the inversion of *SUI1*-*SLA2*-*MAT*α1 previously described [56] is restricted to FEL isolates (Figure 3). The *MAT***a** locus has the same structure in both SEL and FEL isolates, with a single *MAT* gene (*MAT***a**2) between *SLA2* and *GNEAS1* (Figure 3). 

**Figure 4:**
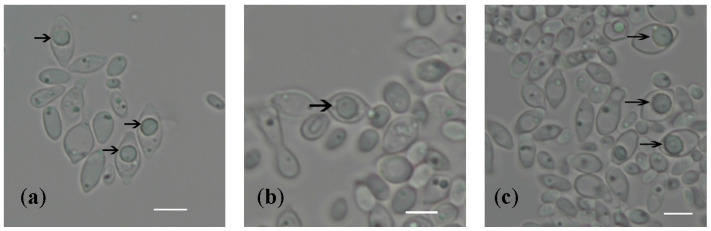
Formation of ascospores of *Hanseniaspora menglaensis* strains. (**a**) CBS 16921 ascospores (indicated with arrows) in asci; (**b**) CBS 18246 ascospore (indicated with arrow) in ascus; (**c**) CBS 18247 ascospores (indicated with arrows) in asci. Scale bar = 5 µm.

Some isolates of the *Hanseniaspora* species are diploid, and contain both *MAT***a** and *MAT*α loci (e.g., *H. vinae*, *H. nectarophila* and *H. hatyaenisis*) (Figure 3). These isolates are likely to be heterothallic, with one *MAT* locus originating from one parent and the other from a second parent. In other isolates, only one *MAT* locus has been identified: only *MAT*α in *H. osmophila*, *H. occidentalis*, *H. menglaensis*, *H. jakobsenii*, and *H. lindneri*, and only *MAT***a** in *H. gamundiae*, *H. mollemarum* and *H. valbyensis* (Figure 3). These are likely to be haploid and heterothallic species, and the missing *MAT* locus may be present in other isolates of the same species. For example, *MAT***a** and *MAT*α have been identified in different isolates of *H. pseudoguilliermondii* (Figure 3; [56]). However, it is also possible that the isolates are diploid, and the second *MAT* locus has not been identified in the genome assemblies. For example, the genome of *H. vinae* TO2/19AF was assembled twice (from the same data), and in one iteration, the *MAT***a** locus was assembled, and in the second, the *MAT*α locus was assembled (Figure 3). In addition, although Chen et al. [13] did not observe ascospores in the Chinese isolate of *H. menglaensis*, ascospores are formed by the Irish isolates (Figure 4). The species is likely to be homothallic as asci with warty ascospores were observed routinely after 7 days for each of the studied strains, CBS 16921, CBS 18246 and CBS 18247, when grown as separate cultures on sporulation medium. The sexual cycle of *H. menglaensis* therefore requires further exploration.

It is notable that the *MAT* locus in *H. opuntiae* appears to have arisen from a recombination between *MAT***a** and *MAT*α, and contains both *MAT***a**2 and *MAT*α1 (Figure 3) (previously described in Saubin et al. [56]). This may be a homothallic species. However, some *H. opuntiae* isolates appear to encode only *MAT***a**2, and hybrids between *H. opuntiae* and *H. pseudoguilliermondii* have been identified [56].

### 3.3. Physiological Analysis

All described *H. menglaensis* isolates have a narrow range of carbon utilisation. Glucose, cellobiose, arbutin, salicin and glucono D-lactone are assimilated (Table 1). Unlike *H. menglaensis* CICC 33364/NYNU 181083, galactose and inulin are assimilated by the three Irish isolates, albeit weakly, with delayed assimilation for inulin (Table 1) [13]. We did not observe growth of the Irish isolates on D-gluconate, which has been reported for *H. menglaenisis* CICC 33364/NYNU 181083 (Table 1) [13]. There are some differences in nitrogen utilisation between the isolates. All isolates assimilate L-lysine, but only *H. menglaenisis* CICC 33364/NYNU 181083 grows on tryptophan (Table 1) [13]. All Irish isolates also use cadaverine and creatine, unlike CICC 33364/NYNU 181083 (Table 1) [13]. Unlike *H. menglaenisis* CICC 33364/NYNU 181083, the Irish isolates do not grow at 30 °C (Table 1) [13].

## 4. Discussion

As of September 2023, 60 *Hanseniaspora* assemblies are publicly available from NCBI GenBank [58]. These include 1 complete, chromosome-level assembly (*H. meyeri*, GCA_030370665.1), 9 contig-level assemblies, and 50 scaffold-level assemblies. We have added another complete chromosome-level assembly for a newly discovered species (*H. menglaensis*), which will facilitate future comparative analysis.

Yeast mitochondrial genomes vary greatly in size, ranging from 18 to more than 105 kb [50]. *H. menglaensis* contains a small mitochondrial genome, similar in size to that of its close relative *H. uvarum* (~19.6 kb and ~18.5 kb, respectively). The *H. uvarum* mitochondrial genome is linear and has identical repeat regions of 3543 bp at each end [51], similar to the mitochondrial genome of *H. meyeri* [59], whereas the *H. menglaensis* mitochondrial genome is circular. *H. meyeri* and *H. uvarum* belong to a different branch than *H. menglaensis* within the FEL (Figure 2), suggesting that there may be a difference in mitochondrial organization between sub-lineages of the FEL clade. However, mitochondrial genome assemblies of other FEL species are needed to confirm this. The mitochondrial genomes from *H. uvarum* and *H. menglaensis* differ in their G+C content (~30% and 24%, respectively). The gene content of *Hanseniaspora* mitochondrial genomes are similar to other *Saccharomycetaceae* species, containing all core components except for NADH ubiquinone oxidoreductase genes [50]. The mitochondrial genes are also short, similar to those in *H. uvarum* [51]. The RNaseP subunit (*rpm1*) is absent from the *H. menglaensis* assembly; however, this element is consistently poorly annotated among yeast species [50]. The ribosomal protein *VAR1* gene is present in the SEL *Hanseniaspora* clade and in the FEL subclade that includes *H. menglaensis*, *H. singularis*, *H. mollemarum*, *H. smithiae*, *H. valbyensis*, and *H. lindneri* (Figure 2). However, *VAR1* is missing from the FEL subclade containing *H. uvarum* [50,51] (Figure 2). *VAR1* is also missing from species in the CTG-Ser1 clade but is present in most other *Saccharomycetaceae* [50,60]. The functional consequence of this gene loss is not clear.

*H. menglaensis* was identified from rotting wood in China [13] and from soil in Ireland, suggesting that it may be a soil saprobic yeast. The genomes of the Irish isolates are highly similar, with a sequence divergence of ~0.0013%. In wild and domestic *S. cerevisiae* populations, sequence divergence between 0.001 and 1.1% has been observed, with an average of 0.5% [61]. Isolates of the human pathogen *Candida albicans* have a divergence of ~0.5% (between isolates of the same clade) and 1.1% (between isolates of different clades) [62]. The sequence divergence in the Irish *H. menglaensis* is surprisingly low, considering that they originated from locations up to 180 km apart and that they belong to a fast-evolving lineage (Figure 2). It is possible that there was a recent genetic bottleneck or a founder effect in the evolutionary history of the Irish population. Comparisons of the whole-genome sequences of the Irish and Chinese isolates (which have not yet been sequenced) may help to address this in the future.

Heterothallic isolates from both the FEL and SEL lineages of *Hanseniaspora* have been described previously [55,56]. *MAT***a** and *MAT*α idiomorphs have been identified in haploid and diploid isolates [55,56] (Figure 3). Each idiomorph encodes only one protein (Mat**a**2 or Matα1, respectively), unlike many other yeast species where the *MAT***a** locus encodes M**ata**1 and Mat**a**2, and *MAT*α encodes Matα1 and Matα2 [52,56]. In *S. cerevisiae,* Mat**a**1 and Matα2 are homeodomain proteins that repress the expression of cell-type specific genes in diploid isolates [52,53,54,63,64]. This dimeric repressor appears to be absent in *Hanseniaspora*. Matα1 regulates the expression of α-specific genes, and (outside the immediate neighbors of *S. cerevisiae*) Mat**a**2 plays a similar role in activating the expression of **a**-specific genes [53,54,55,65,66].

The synteny of the *MAT* locus is generally well conserved in yeast, and it is often adjacent to the *SLA2* gene [52,53,54,55,56]. This pattern is also observed in *Hanseniaspora* species (Figure 3) [56]. Saubin et al. [56] previously identified an inversion around *SUI1-SLA2* in *MATα* idiomorphs in some *Hanseniaspora* isolates. Our analyses show that the rearrangement occurs exclusively in members of the FEL and likely occurred in an ancestor of this lineage (Figure 3). The locus in *H. opuntiae* AWRI 3578, which is probably homothallic, likely arose from a recombination between a *MAT***a** and a rearranged *MAT*α locus (Figure 3).

All three sequenced *H. menglaensis* isolates contain only a *MAT*α locus, consistent with a haploid and heterothallic structure. Chen et al. [13] did not observe ascospore formation in the Chinese isolate (CICC 33364/NYNU 181083), which also suggests that they are haploid [13]. However, ascospores are formed by the Irish isolates (Figure 4). In addition, 24–40 heterozygous sites were identified in the three isolates. It therefore remains possible that the genomes are highly homozygous diploids and that *MAT***a** is present but was not assembled. As the mechanisms of mating and sporulation in *Hanseniaspora* are poorly understood, further investigation is required to underline the processes at work.

The physiology of all five *H. menglaensis* isolates is similar (Table 1) [13], but there are some differences. For example, all isolates assimilate nitrogen from lysine, but only the Chinese isolate uses tryptophan and only the Irish isolates use cadaverine (Table 1). All are signatures of association with plant material, where these nitrogen sources are commonly found. Other differences include the ability of the Chinese isolate to grow at temperatures up to 30 °C, which may indicate an adaptation to different locations. Chen et al. [13] suggest that the ability to assimilate D-gluconate is a distinguishing factor between *H. menglaensis* and *H. lindneri*. However, the Irish isolates cannot assimilate D-gluconate (Table 1). We do note that an inability to metabolise ethylamine distinguishes all five *H. menglaensis* isolates from *H. lindneri* [13,57].

## Figures and Tables

**Table 1:** Biochemical characteristics of *Hansenisaspora menglaensis* and *Hansenisaspora linderi*. Characteristics of *H. menglaensis* CICC 33364/NYNU 181083 and *H. linderi* are taken from previous studies [1,13,57].

Species	*H. menglaensis* CBS 16921	*H. menglaensis* CBS 18246	*H. menglaensis* CBS 18247	*H. menglaensis* CICC 33364/NYNU 181083	*H. lindneri*
Glucose	+	+	+	+	+
Cellobiose	+	+	+	+	d
Arbutin	+	+	+	+	d
Salicin	+	+	+	+	d
Glucono D-lactone	+	+	+	+	d
D-Galactose	w	w	w	-	-
Inulin	dw	dw	dw	-	-
Soluble Starch	dw	dw	dw	n	n
D-Gluconate	-	-	-	+	-
Lysine	+	+	+	+	+
Ethylamine	-	-	-	-	+
Cadaverine	+	+	+	-	+
Creatine	+	+	+	-	n
Tryptophan	-	-	-	+	n
30 °C	-	-	-	+	+

+, positive; -, negative; w, weak; d, delayed; dw, delayed and weak; n, not available.

## Data Availability

Whole-genome sequencing data for strains CBS 18246 (SRR24099574), CBS 18247 (SRR24099573), and CBS 16921 (SRR24099575/SRR24099572), and the genome assembly for CBS 16921 (CP128838-CP128845) are available at NCBI GenBank under BioProject PRJNA950348.

## Appendix A

**Table A1:** Comparison of ITS and D1/D2 regions to CBS 16921.

	ITS		D1/D2	
Isolate	% Identity	Accession	% Identity	Accession
CBS 16921 *	100	OR939358	100	SRR24099575
CBS 18247 *	99.3	OR939360	99.8	SRR24099573
CBS 18246 *	99.3	OR939359	99.8	SRR24099574
CICC33364	99	MK682803	99.3	MK682799
NYNU 181083	98.9	OQ168353	99.3	OQ168352
*H. lindneri* *	96.7	GCA_019649525	98.4	GCA_019649525
*H. valbyensis*	95.8	KY103578	97.9	KY107858

* sequences derived from whole-genome assembly.

**Table A2:** Comparison of average nucleotide identity (ANI) of CBS 16921 to available *Hanseniaspora* genomes.

Isolate	OrthoANI Value	Average Aligned Length	Query Coverage	Subject Coverage	Subject Length
*H. menglaensis* CBS 16921	100	9,523,740	1	1	9,553,320
*H. menglaensis* CBS 18246	99.96	7,358,704	0.77	0.78	9,432,960
*H. menglaensis* CBS 18247	99.96	6,908,493	0.72	0.74	9,348,300
*H. valbyensis* NRRL Y-1626	76	2,853,907	0.3	0.3	9,664,500
*H. smithiae* CRUB 1602	75.87	3,063,016	0.32	0.33	9,319,740
*H. lindneri* CBS 285	75.5	2,340,075	0.24	0.22	10,647,780
*H. singularis* ZIM 2326	74.87	2,343,548	0.25	0.27	8,784,240
*H. mollemarum* CBS 15034	74.59	2,715,924	0.28	0.3	8,941,320
*H. hatyaiensis* ZIM 2327	74.29	1,621,195	0.17	0.17	9,581,880
*H. uvarum* CBA6001	74.14	1,723,829	0.18	0.19	8,958,660
*H. thailandica* ZIM 2325	74.14	1,655,772	0.17	0.18	9,229,980
*H. opuntiae* AWRI3578	74.04	1,434,013	0.15	0.16	8,820,960
*H. jakobsenii* ZIM 2603	73.96	1,648,240	0.17	0.12	13,340,580
*H. guilliermondii* NRRL Y-1625	73.94	1,582,303	0.17	0.18	8,974,980
*H. meyeri* NRRL Y-27513	73.94	1,462,711	0.15	0.15	9,746,100
*H. nectarophila* CBS 13383	73.79	1,636,636	0.17	0.18	9,218,760
*H. clermontiae* NRRL Y-27515	73.77	1,487,723	0.16	0.17	8,667,960
*H. pseudoguilliermondii* ZIM 213	73.42	1,509,933	0.16	0.17	8,747,520
*H. lachancei* NRRL Y-27514	73.39	1,449,502	0.15	0.16	8,821,980
*H. occidentalis* CBS 6783	72.26	712,638	0.07	0.06	11,567,820
*H. osmophila* AWRI3579	71.96	647,824	0.07	0.06	11,449,500
*H. gamundiae* CRUB 1928	71.73	597,327	0.06	0.06	10,006,200
*H. vineae* T02/19AF	71.58	689,535	0.07	0.06	11,293,440
